# Special Issue: Advances in Electrochemical Energy Materials

**DOI:** 10.3390/ma13040844

**Published:** 2020-02-13

**Authors:** Shiqi Li, Zhaoyang Fan

**Affiliations:** 1 College of Electronic Information, Hangzhou Dianzi University, Hangzhou 310018, China; sqli@hdu.edu.cn; 2 Department of Electrical and Computer Engineering and Nano Tech Center, Texas Tech University, Lubbock, TX 79409, USA

**Keywords:** lithium ion batteries, supercapacitors, electrode materials, nanostructure, electrochemical energy storage

## Abstract

Electrochemical energy storage is becoming essential for portable electronics, electrified transportation, integration of intermittent renewable energy into grids, and many other energy or power applications. The electrode materials and their structures, in addition to the electrolytes, play key roles in supporting a multitude of coupled physicochemical processes that include electronic, ionic, and diffusive transport in electrode and electrolyte phases, electrochemical reactions and material phase changes, as well as mechanical and thermal stresses, thus determining the storage energy density and power density, conversion efficiency, performance lifetime, and system cost and safety. Different material chemistries and multiscale porous structures are being investigated for high performance and low cost. The aim of this Special Issue is to report the recent advances of materials used in electrochemical energy storage that encompasses supercapacitors and rechargeable batteries.

Electrochemical energy materials are used for electrochemical energy storage or conversion. Broadly speaking, these include materials used in batteries and supercapacitors, as well as electrocatalysts to produce new fuels. In this Special Issue, we focus on those in lithium-ion batteries (LIBs) and supercapacitors, particularly the electrode active materials and their structure that must be capable of supporting multitude of coupled physicochemical processes as well as mechanical and thermal stresses. They directly determine the overall performance of the energy storage, including ultimate energy and power densities, lifetime, and system cost and safety. 

The commercialized LIB now uses graphitic carbon as its anode, which has a theoretical capacity of 372 mAh g^−1^ based on Li^+^ intercalation between the graphite layers. Many other materials can form alloys with lithium and thus provide much higher capacity. These materials, however, generally suffer from a large volume change during the alloying-dealloying process, leading to quick fading of the anode capacity. Nanostructure engineering is a practical approach to release stress, thus minimizing electrode material pulverization. In contrast to the anode, the overall performance of a LIB nowadays is largely constrained by its cathode, which has only about half the specific capacity of the graphite anode. The cathode is also the most expensive and the heaviest component in an LIB. Therefore, increasing the cathode specific capacity is crucial for better and cheaper LIBs. In this regard, Li-rich manganese-based layered oxides with the chemical formula xLi_2_MnO_3_·(1−x)LiMO_2_, where M represents transition metal elements, with its capacity up to 300 mAh g^−1^ has drawn considerable attention. Other cathode materials also still have enough room for further improvement of their performance. In addition to electrodes, developing solid electrolytes in substitution of the liquid electrolyte and the separator for producing solid-state LIBs is another active area. The solid-state LIBs will address the safety issue related to the organic solvent-based electrolyte and also have potential to increase the energy density.

LIBs can store a large amount of energy, but the slow kinetics in the electrochemical process restrain the rate of energy storing and releasing, or the charging current rate and output power density. There are plenty of applications that require high-power and high-rate energy storage with much long cycle lifetime where LIBs cannot meet the demand. Electrochemical supercapacitors can fit into these needs very well. 

Conventional supercapacitors are those storing charges electrostatically in the electrical double layer formed on an inertial carbon surface, or electrical double layer capacitors (EDLCs). They offer a high-power density and a long cycle lifetime, but with an energy density less than 10 Wh kg^−1^, more than 20 folds smaller than that of LIBs. Therefore, pseudocapacitors are being actively investigated to store charges in a surface-related reversible faradic redox reaction, thus offering much larger capacitance than EDLCs to bridge the energy density gap from batteries. On the other end of the spectrum, EDLC has a frequency response limited to 1 Hz or so, mainly caused by its mesoporous carbon electrode structure. Developing ultrafast EDLCs that can effectively work at hundreds and even kHz domain will broaden the function of EDLCs into the area of filtering capacitors and therefore, is also attracting much attention.

The Special Issue “Advances in Electrochemical Energy Materials” was proposed to present recent developments in this active field. The twelve articles included touch different aspects of materials for electrochemical energy storage, which are introduced in the following.

The main theme of material research for LIBs is centred on the high capacity anode and cathode. The article by Zhang et al. [1] reported on nanotube structured ZnS as the anode of LIBs. ZnS is considered as a promising alternative to graphitic carbon due to its much higher theoretical capacity of 962.3 mAh g^−1^, but its large volume change during the charging and discharging process hinders its practical application. Nanotube structured ZnS anode was therefore prepared using ZnO nanotubes as a sacrificial template, expecting that the radially and longitudinally expansion of nanotubes could mitigate the stress and thus improving the electrode stability. A high initial capacity and reasonable cycling stability were demonstrated for this ZnS anode structure.

Developing high-capacity and low-cost cathode materials for LIBs has attracted considerable attention. This is particularly true to Li-rich layered oxides. However, these materials suffer from severe voltage and capacity fading caused by continuous phase transition from layered phase to spinel or others during the repeated Li^+^ extraction/insertion process. Composition control to maintain the structural stability is hence crucial for cathodes with prolonged structural integrity and enhanced electrochemical performance. Chang et al. [2] reported a study of layered Li_1.27_Cr_0.2_Mn_0.53_O_2_ powders with mesoporous structure synthesized by a nanomilling-assisted solid-phase method. The fabricated cathode delivered a capacity close to its theoretical value with good capacity retention after 100 charging-discharging cycles. No transformation of the layered crystal structure was confirmed. Two papers from Chen’s group [3,4] presented their studies on the Li-rich manganese-based layered oxides. It was found that a high nickel content in the layered phase could stabilize the structure and alleviate the voltage and capacity attenuation [3]. This was explained that some Ni^2+^ ions occupy the Li^+^ ion sites and this cation doping improves the structural stability by supporting the Li slabs and reducing tension of neighboring oxygen layers during the delithiation process. The preferential reduction of Ni^4+/2+^ also maintains the average oxidation state of Mn above 3^+^, effectively improving structural durability. Composition uniformity is another crucial parameter which might be related to the synthesis method [4]. The sol–gel and the oxalate co-precipitation synthesis methods were subsequently compared based on the microstructure, element distribution, and electrochemical performance of the prepared manganese oxides with a high nickel content. The uniform element distribution in samples synthesized by the oxalate co-precipitation method further contributed to the stability of the layered structure. 

Other than manganese-based, Li-rich molybdenum-based layered oxides was also attractive. Yu et al. [5] investigated Co doping in Li_2_MoO_3_ to improve its structure stability and electronic conductivity. Their results showed that an appropriate amount of Co ions can be introduced into the Li_2_MoO_3_ lattices and electrochemical tests revealed that Co-doping can significantly improve the electrochemical performances of the Li_2_MoO_3_ materials. 

In addition to these new cathode materials, a further study of the conventional olivine-type LiFePO_4,_ was also carried out, aiming to reduce the manufacturing cost and minimize pollutants generation. Liu et al. [6] developed a green route to produce the LiFePO_4_/C composite, which showed a uniform carbon coating on LiFePO_4_ nanoparticles, with effectively improved conductivity and enhanced Li^+^ ion diffusion. Consequently, LIBs using the synthesized composite as cathode materials exhibited superior performance, especially at high rates.

Besides experimental test of electrode stability, simulation-driven selection of electrode materials based on mechanical performance during lithiation/delithiation process was also studied. Sarkar et al. [7] developed a model to determine particle deformation and stress fields by combining the stress equilibrium equations with the Li^+^ electrochemical diffusion. It was applied to derive five merit indices to reflect the mechanical stability of electrode materials. The authors further suggested ways for the selection and optimal design of electrode materials to improve their mechanical performance.

Solid-state LIBs are being pursued as the next-generation energy storage technology to provide high safety and high energy density. For this technology, the solid electrolyte with high ionic conductivity and electrochemical stability is the most crucial component. Ji et al. [8] studied the synthesis of Nb-doped lithium garnet Li_7_La_3_Zr_2_O_12_ (LLZNO) as a high ionic conductive solid electrolyte. Submicron size LLZNO powder was prepared using a solid-state reaction and an attrition milling process, followed by sintering at a relatively low temperature for a short time. The properties of the synthesized LLZNO and its performance in a solid-state LIB were reported.

This Special Issue also includes several papers presenting the research on electrode materials and structures for supercapacitors, particularly for pseudocapacitors. The electrode materials for pseudocapacitors commonly include transition metal oxides, nitrides, and sulfides, among others. Since the pseudocapacitive effect is commonly surface or sub-surface related, a large surface area of these compounds is crucial for achieving a high specific capacitance. Their generally low conductivity is another issue to be addressed for high-rate and high-power performance. These compounds, therefore, are commonly synthesized into a nanoparticle form anchored on a carbon-based conductive framework. In the work by Sun et al. [9], a biomorphic porous composite was prepared with Mn_3_O_4_ nanocrystals anchored on porous carbon microfiber, with the latter derived from cotton wool. The unique structure resulted in the good cycling stability of the fabricated supercapacitors. Amade et al. [10] reported using graphene nanowalls, which were grown in a plasma-enhanced chemical vapor deposition process, as the framework for manganese dioxide deposition by electrodeposition. More interesting work by Ahmad et al. [11] investigated nanoporous carbon, derived from zeolitic imidazolate framework (ZIF-67) as the support of cobalt sulfide, which was formed through anion exchange sulfidation process from cobalt oxide. A large capacitance of 677 F g^−1^ was obtained.

There is strong interest in developing high-frequency supercapacitors or electrochemical capacitors (HF-ECs) [12] for line-frequency alternating current (AC) filtering in the substitution of bulky aluminum electrolytic capacitors, with broad applications in the power and electronic fields. Edge-oriented vertical graphene networks on 3D scaffolds have a unique structure that offers straightforward pore configuration, reasonable surface area, and high electronic conductivity, thus allowing the fabrication of HF-ECs. Comparatively, highly conductive freestanding cross-linked carbon nanofibers, derived from bacterial cellulose in a rapid plasma pyrolysis process can also provide a large surface area but are free of rate-limiting micropores, and are another good candidate for HF-ECs. Li et al. [12] summarized the recent advances in this field with emphasis on their contributions in the study of these materials and their electrochemical properties including preliminary demonstrations of HF-ECs for AC line filtering and pulse power storage applications.
